# Supplementary material for: 4-aminopyridine attenuates inflammation and apoptosis and increases angiogenesis to promote skin regeneration following a burn injury in mice
Source: Cell Death Discov. 2024 Oct 4;10:428. doi: 10.1038/s41420-024-02199-6 (PMC11452548; doi:10.1038/s41420-024-02199-6)
Supplement: Supplementary file 1 — SUPPLEMENTAL MATERIAL [file 41420_2024_2199_MOESM1_ESM.docx]

**SUPPLEMENTAL MATERIAL**

**4-aminopyridine attenuates inflammation and apoptosis and increases angiogenesis to promote skin regeneration following a burn injury in mice**

Rahul, V. G.^1#^, Govindaraj Ellur^1#^, Amir A. Gaber^1^, Prem Kumar Govindappa^1*^, and John C. Elfar^1*^

^1^Department of Orthopaedics and Sports Medicine, University of Arizona College of Medicine, Tucson, AZ, 85724, USA.

#These authors contributed equally to this work.

*These authors share corresponding authorship.

**Correspondence:**

**Prem Kumar Govindappa**

email: pkgovindappa@gmail.com

**John C. Elfar**

email: openelfar@gmail.com

**
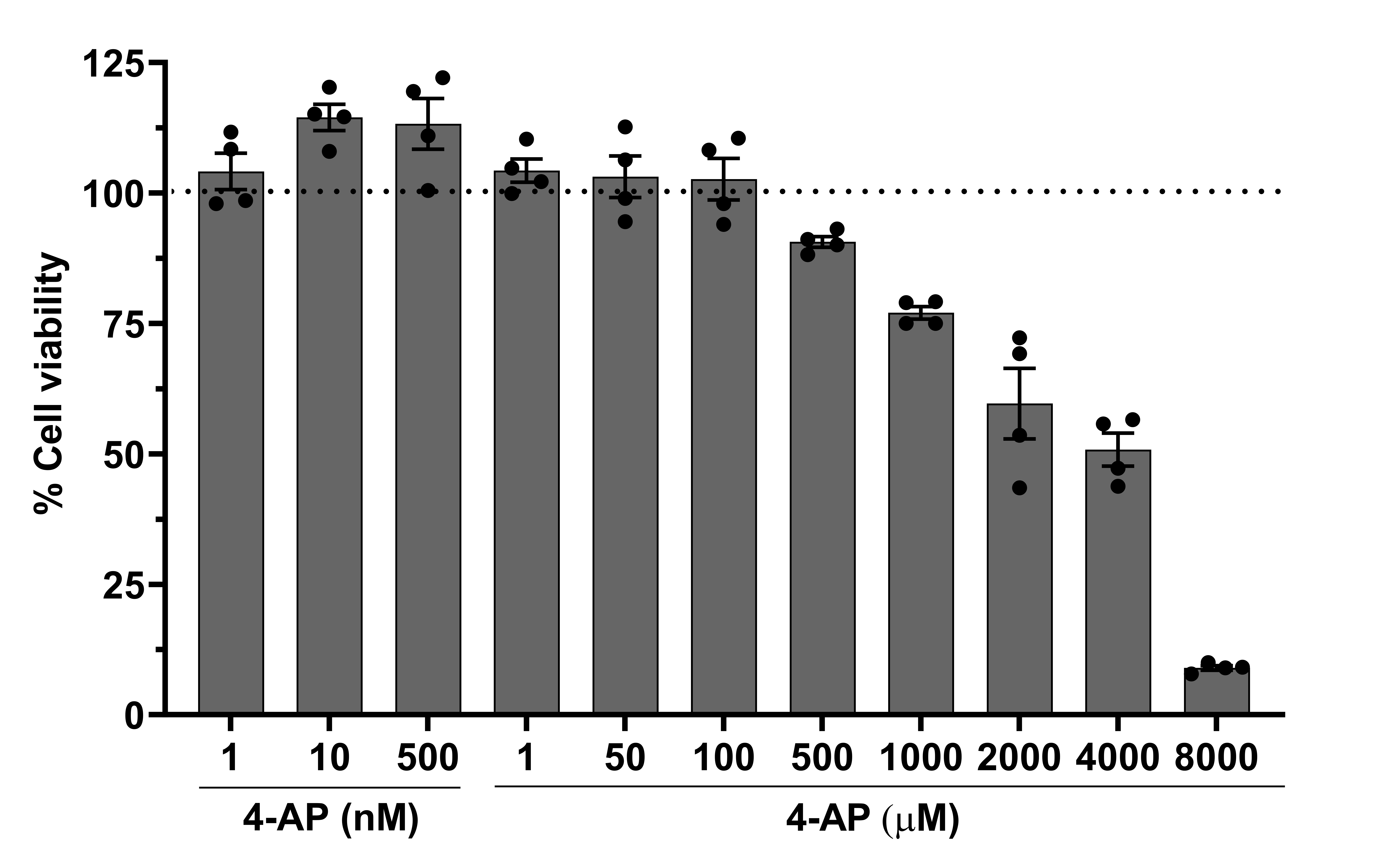
**

**Supplementary Fig. S1. 4-AP effects on BMMØs cell viability by MTT assay.** 4-AP was nontoxic to BMMØs until 100 μM concentration and it showed toxic effect in higher concentrations (500 to 8000 μM) at 24 hr. Untreated cells were used as controls to calculate the % cell viability. n = 4/ concentrations.

**
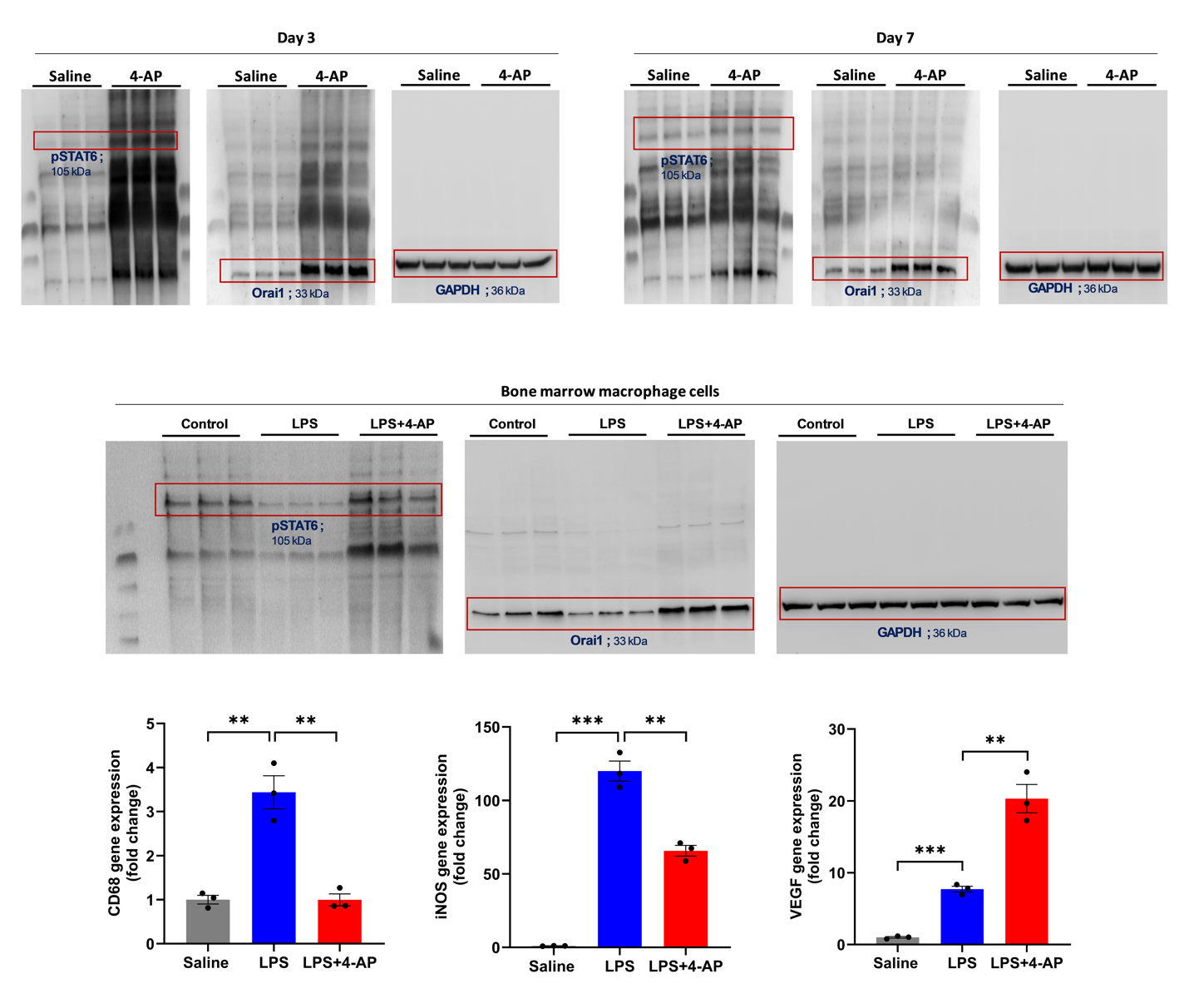
**

**Supplementary Fig. S2. 4-AP attenuated pro-inflammation and accelerated anti-inflammatory effects in BMMØs under LPS stress conditions.** qRT-PCR data shows that 4-AP treatment significantly attenuated pro-inflammatory genes (CD68 and iNOS and augmented anti-inflammatory/angiogenic genes (VEGF) in BMMØs under LPS stress conditions. n = 3 per group.

**Supplementary Table S1. Primers used for gene expression analysis**

| **Gene** | **Primer sequence (5ˈ-3ˈ)** | |
| --- | --- | --- |
|  | **Forward** | **Reverse** |
| **IL1β** | GCAACTGTTCCTGAACTCAACT | ATCTTTTGGGGTCCGTCAACT |
| **TNFα** | GACGTGGAACTGGCAGAAGAG | TTGGTGGTTTGTGAGTGTGAG |
| **ARG-1** | CTCCAAGCCAAAGTCCTTAGAG | AGGAGCTGTCATTAGGGACATC |
| **CD206** | CTCTGTTCAGCTATTGGACGC | CGGAATTTCTGGGATTCAGCTTC |
| **IL10** | CCCATTCCTCGTCACGATCTC | TCAGACTGGTTTGGGATAGGTTT |
| **VEGF** | GCACATAGAGAGAATGAGCTTCC | CTCCGCTCTGAACAAGGCT |
| **CD31** | ACGCTGGTGCTCTATGCAAG | TCAGTTGCTGCCCATTCATCA |
| **eNOS** | GGCTGGGTTTAGGGCTGTG | CTGAGGGTGTCGTAGGTGATG |
| **BAX** | TGAAGACAGGGGCCTTTTTG | AATTCGCCGGAGACACTCG |
| **BCL2** | GTCGCTACCGTCGTGACTTC | CAGACATGCACCTACCCAGC |
| **Caspase-9** | TCCTGGTACATCGAGACCTTG | AAGTCCCTTTCGCAGAAACAG |
| **Caspase-3** | TGGTGATGAAGGGGTCATTTATG | TTCGGCTTTCCAGTCAGACTC |
| **K10** | TCATGTAGGGAGCAACATCCT | AGTAAGACAAGGCGCATCTTC |
| **K14** | GTTTTCGGCCTCTCGCTAGTT | CTGTTCCCGGTCTTGAACC |
| **TGFβ** | AGACCACATCAGCATTGAGTG | GGTGGCAACGAATGTAGCTGT |
| **Vimentin** | CGGCTGCGAGAGAAATTGC | CCACTTTCCGTTCAAGGTCAAG |
| **αSMA** | GACAGGGCAATCACCGTCTTC | CGAGAGCGCAGATTTTCCTCA |
| **FGF** | TAGCCTGATCCGACAGAAGC | GGCAGAACAGTTTGGTGACG |
| **Col-I** | GCTCCTCTTAGGGGCCACT | CCACGTCTCACCATTGGGG |
| **Col-III** | AGATGAGGCGGAAACTCAAGT | AGCCCTTTAGGAAGAGGTGTT |
| **MMP9** | CTGGACAGCCAGACACTAAAG | CTCGCGGCAAGTCTTCAGAG |
| **MMP3** | ACATGGAGACTTTGTCCCTTTTG | TTGGCTGAGTGGTAGAGTCCC |
| **Orai1** | GATCGGCCAGAGTTACTCCG | TGGGTAGTCATGGTCTGTGTC |
| **STAT6** | CTCTGTGGGGCCTAATTTCCA | CATCTGAACCGACCAGGAACT |
| **CD68** | TGTCTGATCTTGCTAGGACCG | GAGAGTAACGGCCTTTTTGTGA |
| **iNOS** | GTTCTCAGCCCAACAATACAAGA | GTGGACGGGTCGATGTCAC |
| **GAPDH** | TGGATTTGGACGCATTGGTC | TTTGCACTGGTACGTGTTGAT |

**UNCUT WESTERN BLOT IMAGES**

**Fig. 2. 4-AP attenuated pro-inflammation and increased anti-inflammatory effects following skin burn.**

**
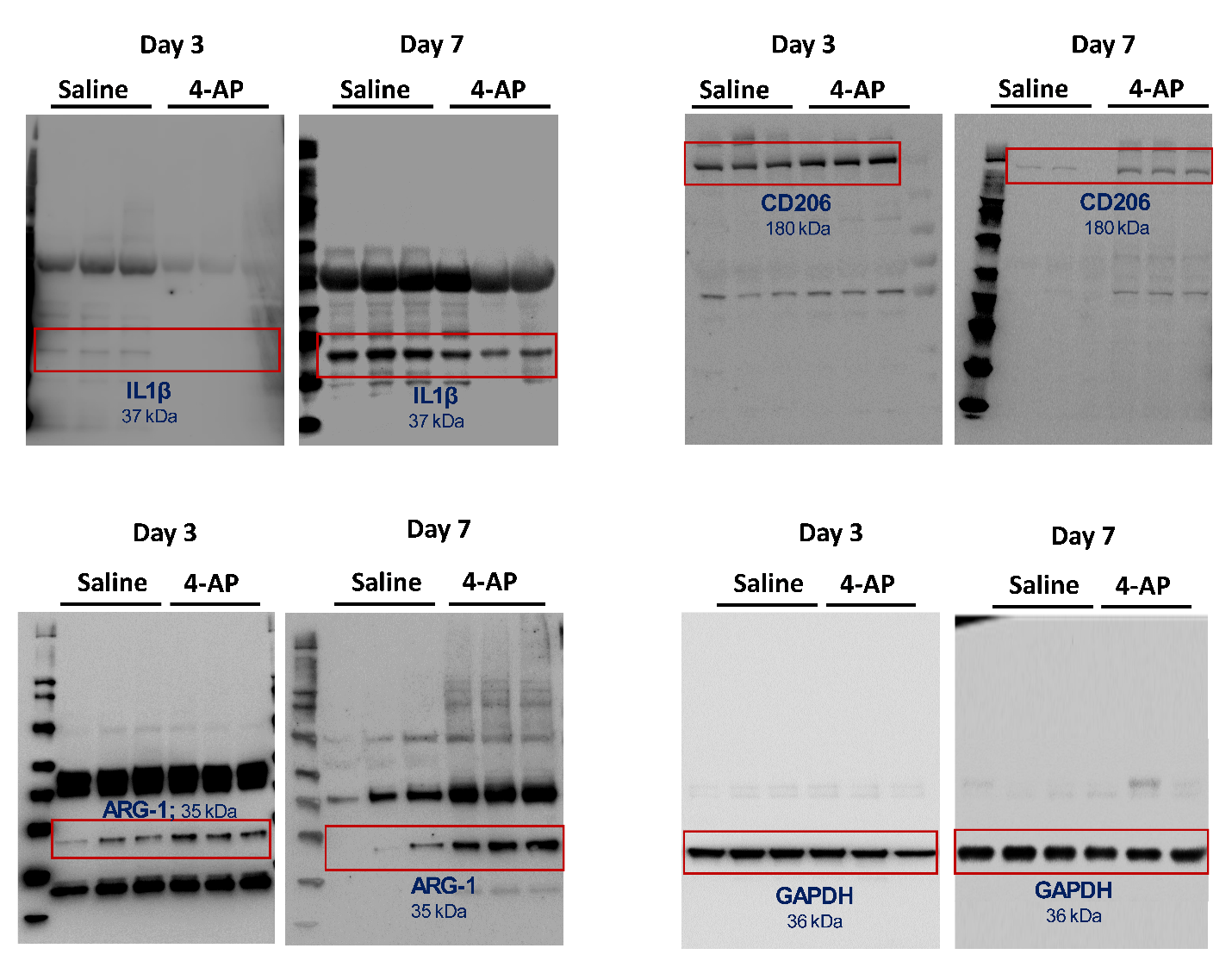
**

**Fig. 3. 4-AP augmented macrophage reparative function via Orai1 calcium channel signaling.**

**
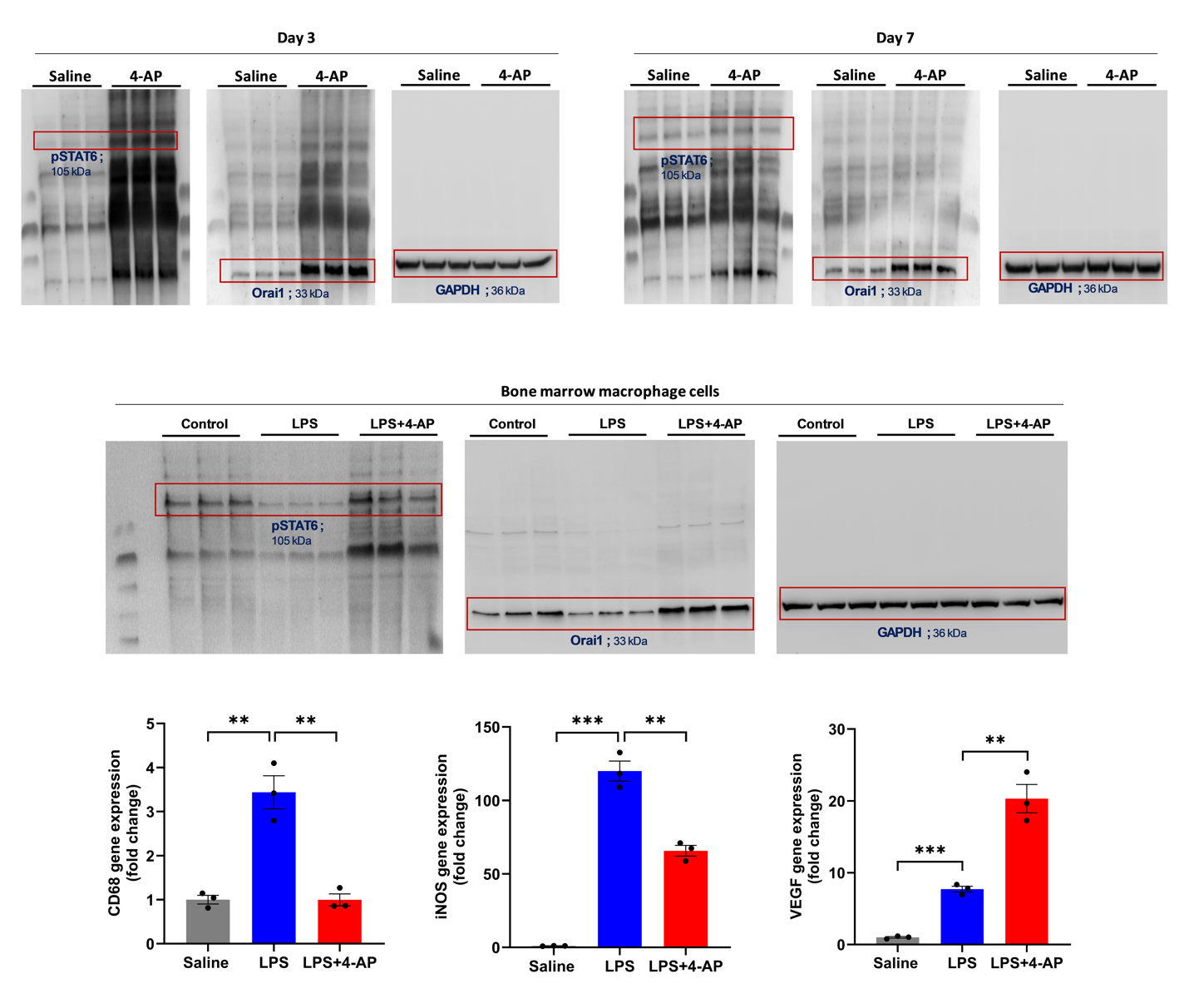
**

**Fig. 4. 4-AP augmented angiogenesis following skin burn.**

**
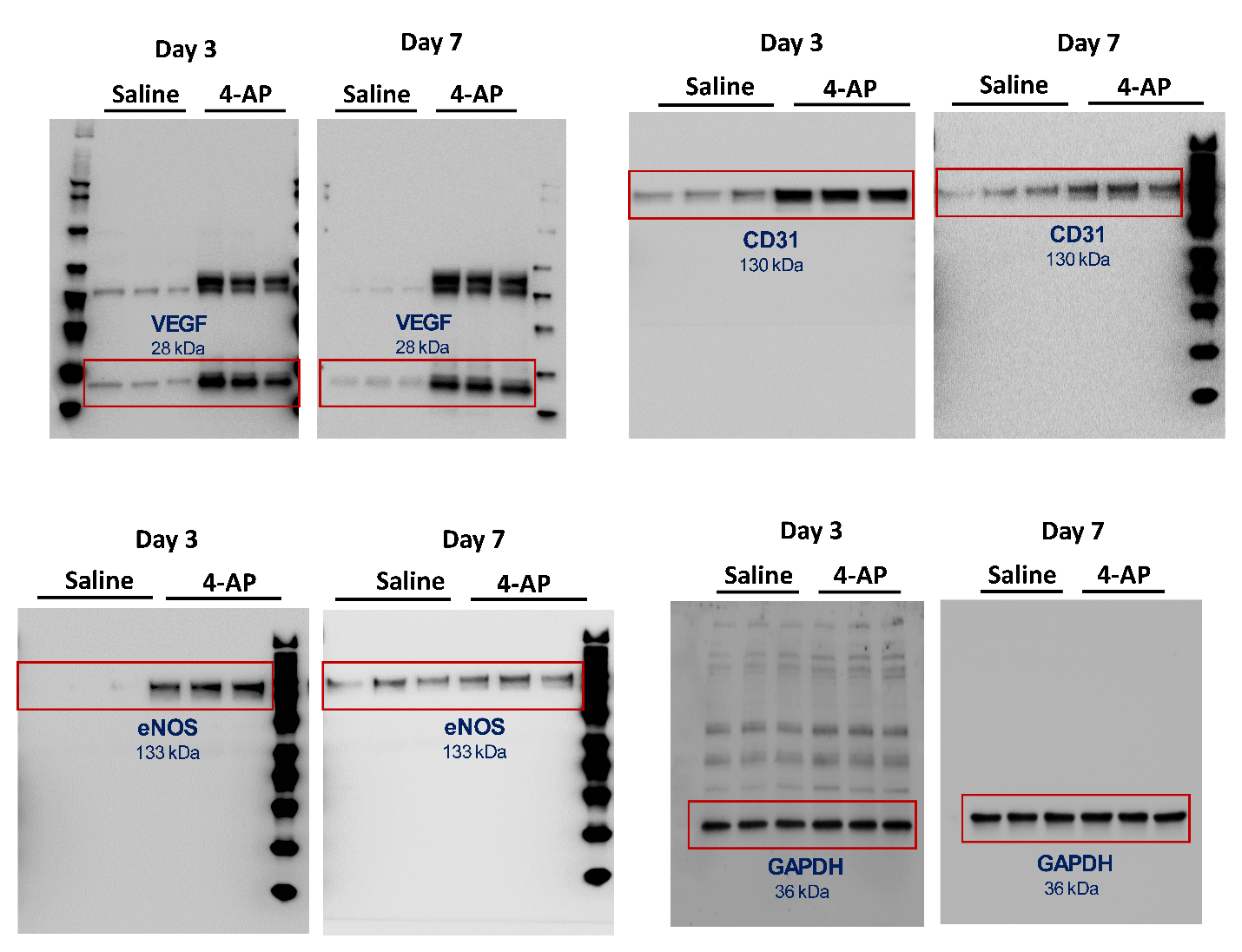
**

**Fig. 5. 4-AP attenuated pro-apoptosis and increased anti-apoptosis effects following skin burn.**

**
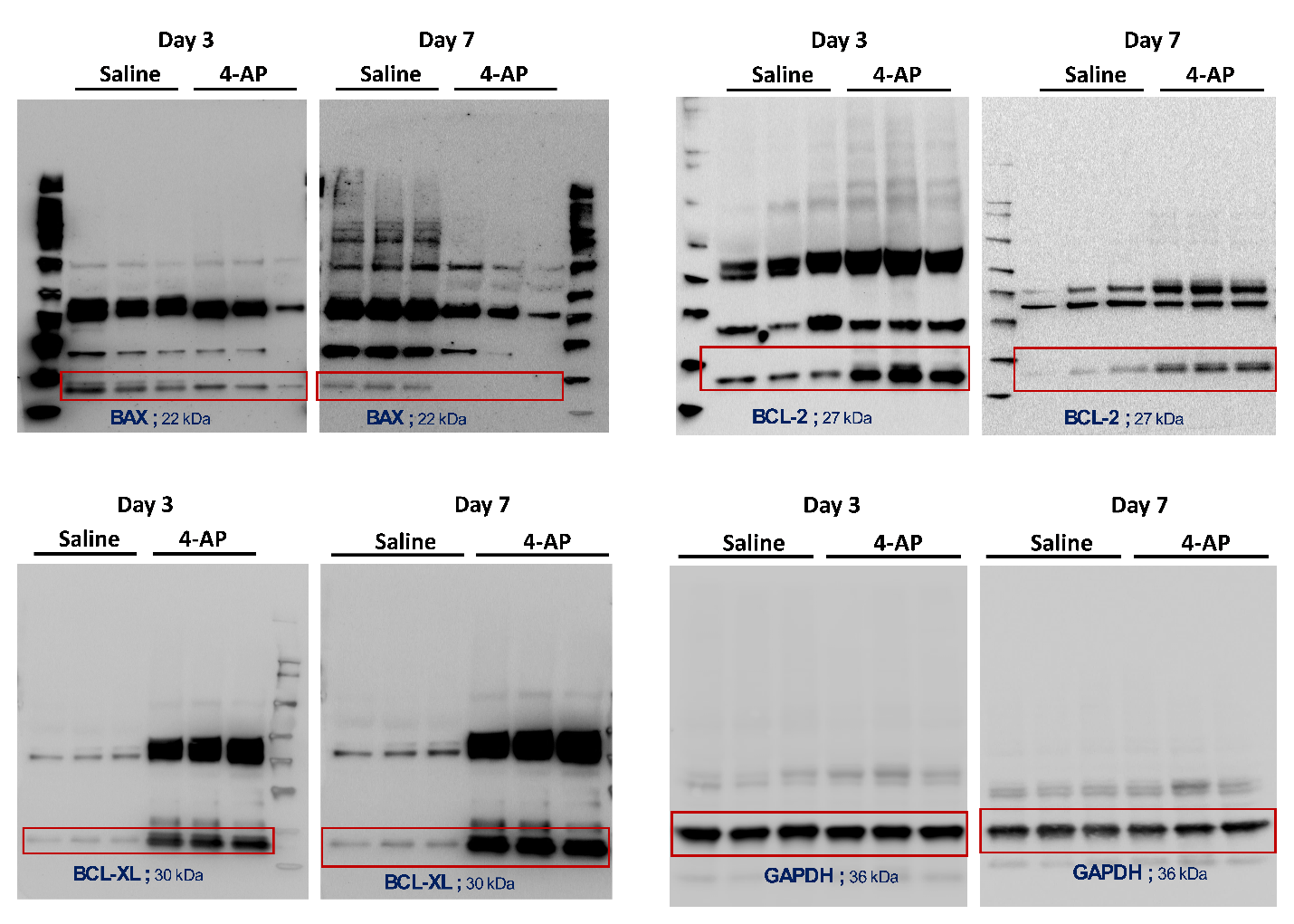
**

**Fig. 6. 4-AP accelerated re-epithelization following skin burn.**

**
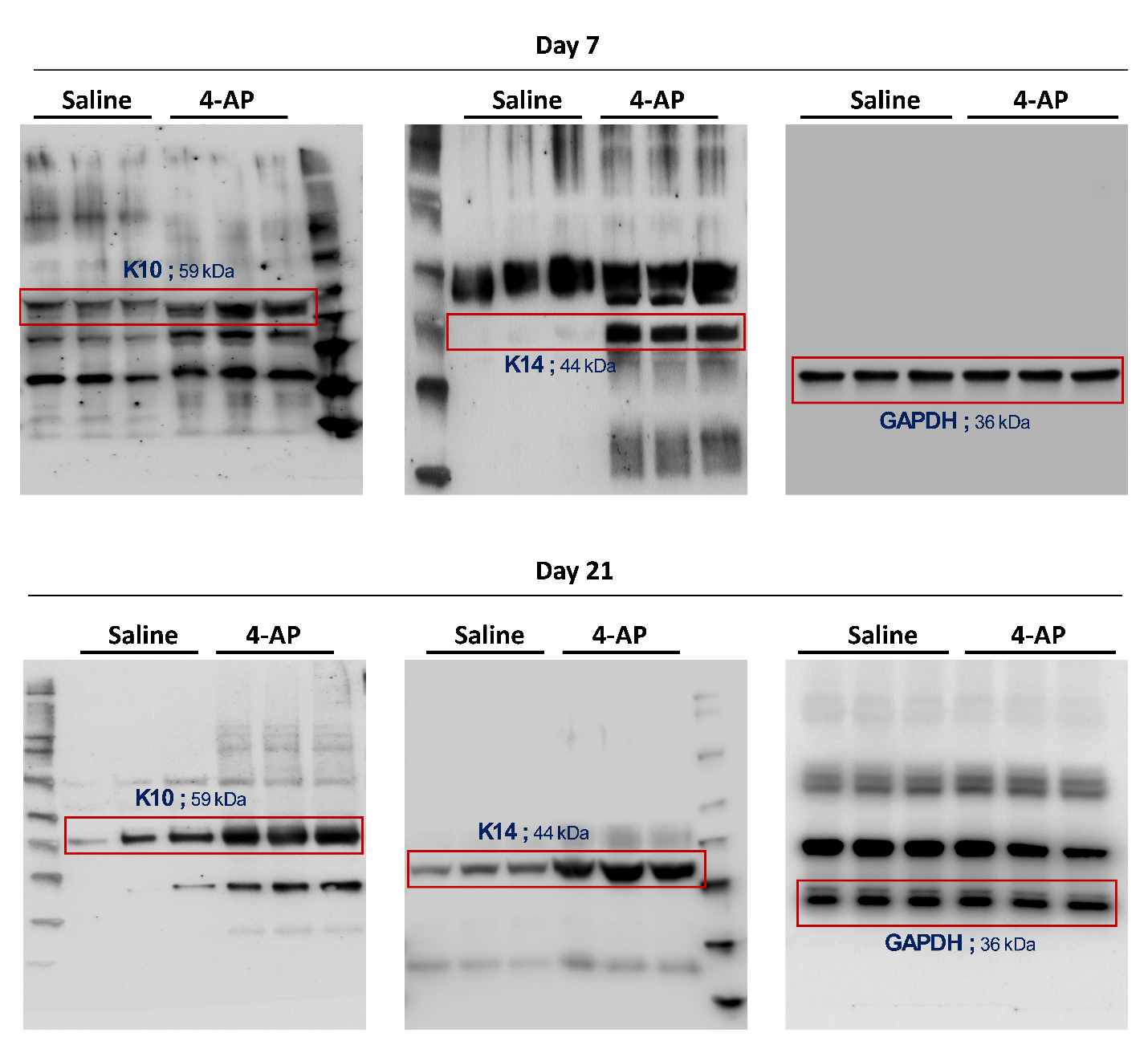
**

**Fig. 7. 4-AP promoted fibroblasts to myofibroblasts transformation following skin wound.**

**
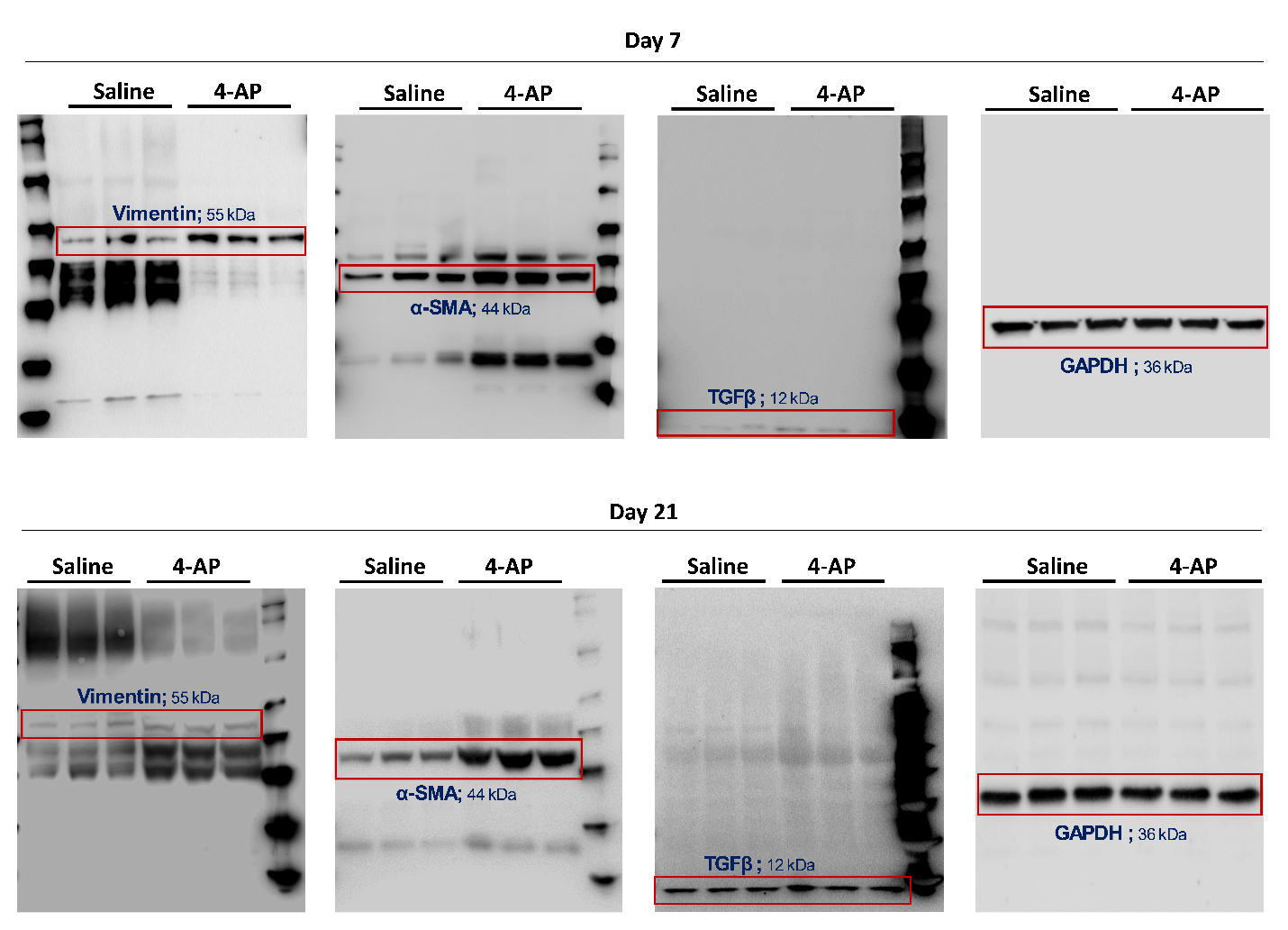
**

**Fig. 8. 4-AP advanced matrix remodeling following skin burn.**

**
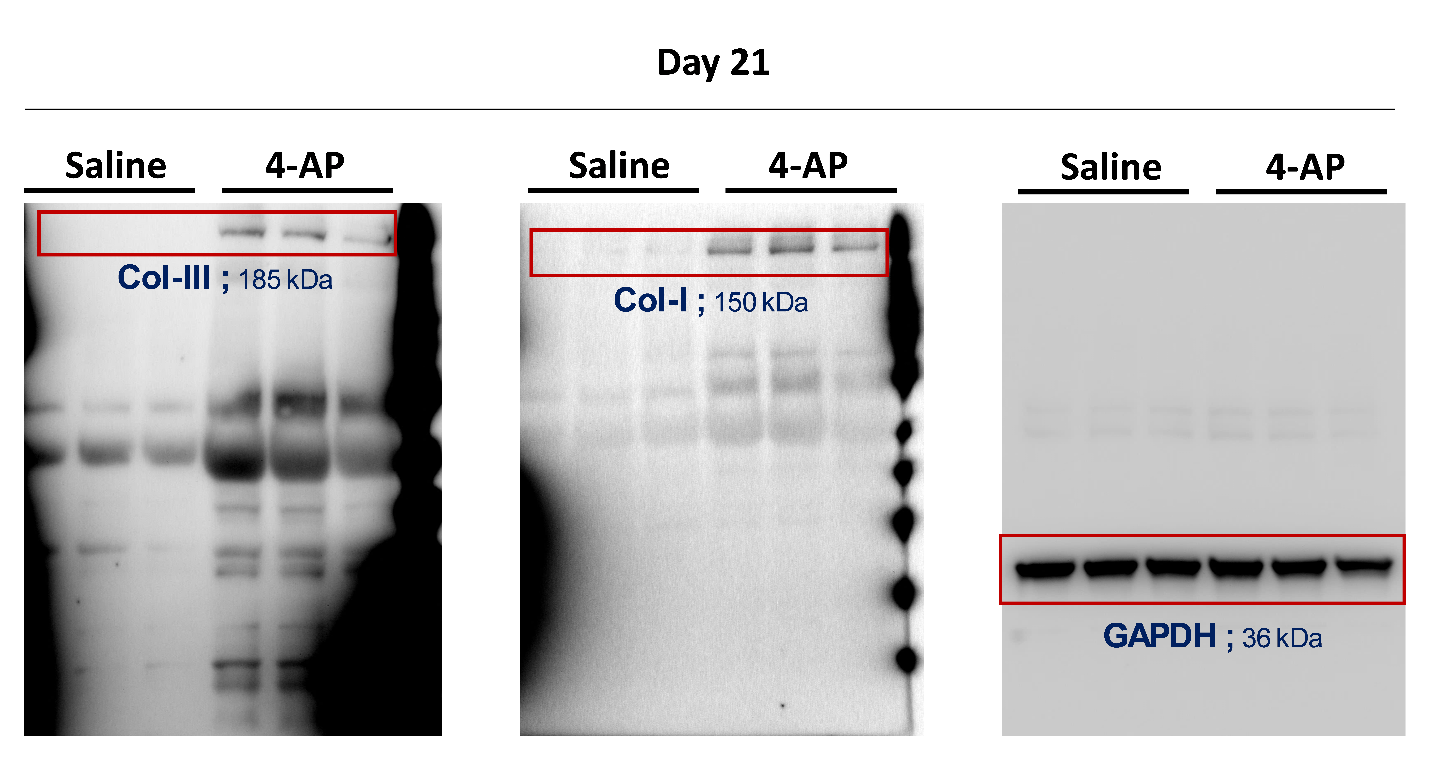
**
