# Supplementary material for: 4-aminopyridine attenuates inflammation and apoptosis and increases angiogenesis to promote skin regeneration following a burn injury in mice
Source: Cell Death Discov. 2024 Oct 4;10:428. doi: 10.1038/s41420-024-02199-6 (PMC11452548; doi:10.1038/s41420-024-02199-6)
Supplement: Supplementary file 2 — UNCROPPED ORIGINAL WESTERN BLOT IMAGES [file 41420_2024_2199_MOESM2_ESM.docx]

**UNCROPPED ORIGINAL WESTERN BLOT IMAGES**

**Fig. 2. 4-AP attenuated pro-inflammation and increased anti-inflammatory effects following skin burn.**

**
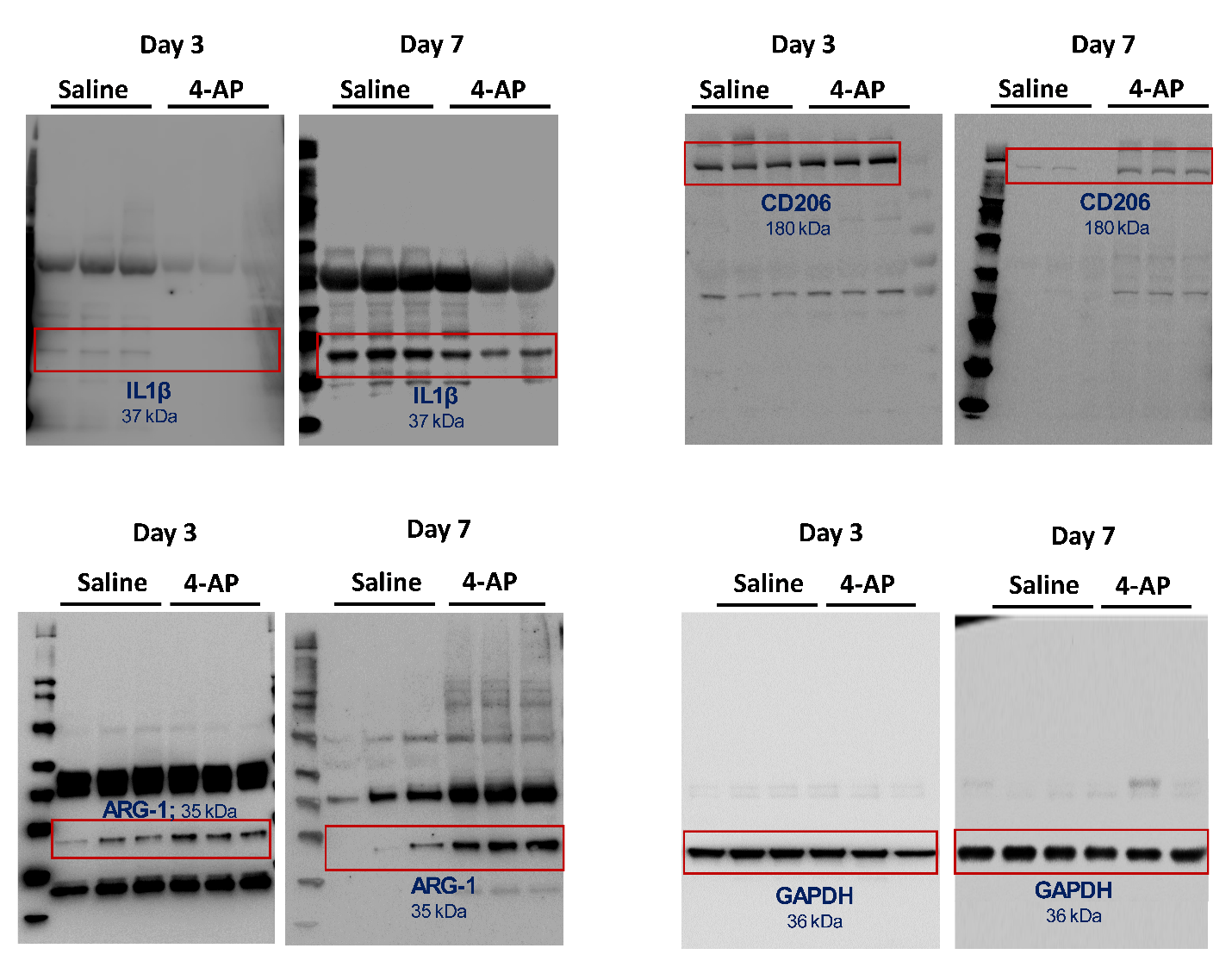
**

**Fig. 3. 4-AP augmented macrophage reparative function via Orai1 calcium channel signaling.**

**
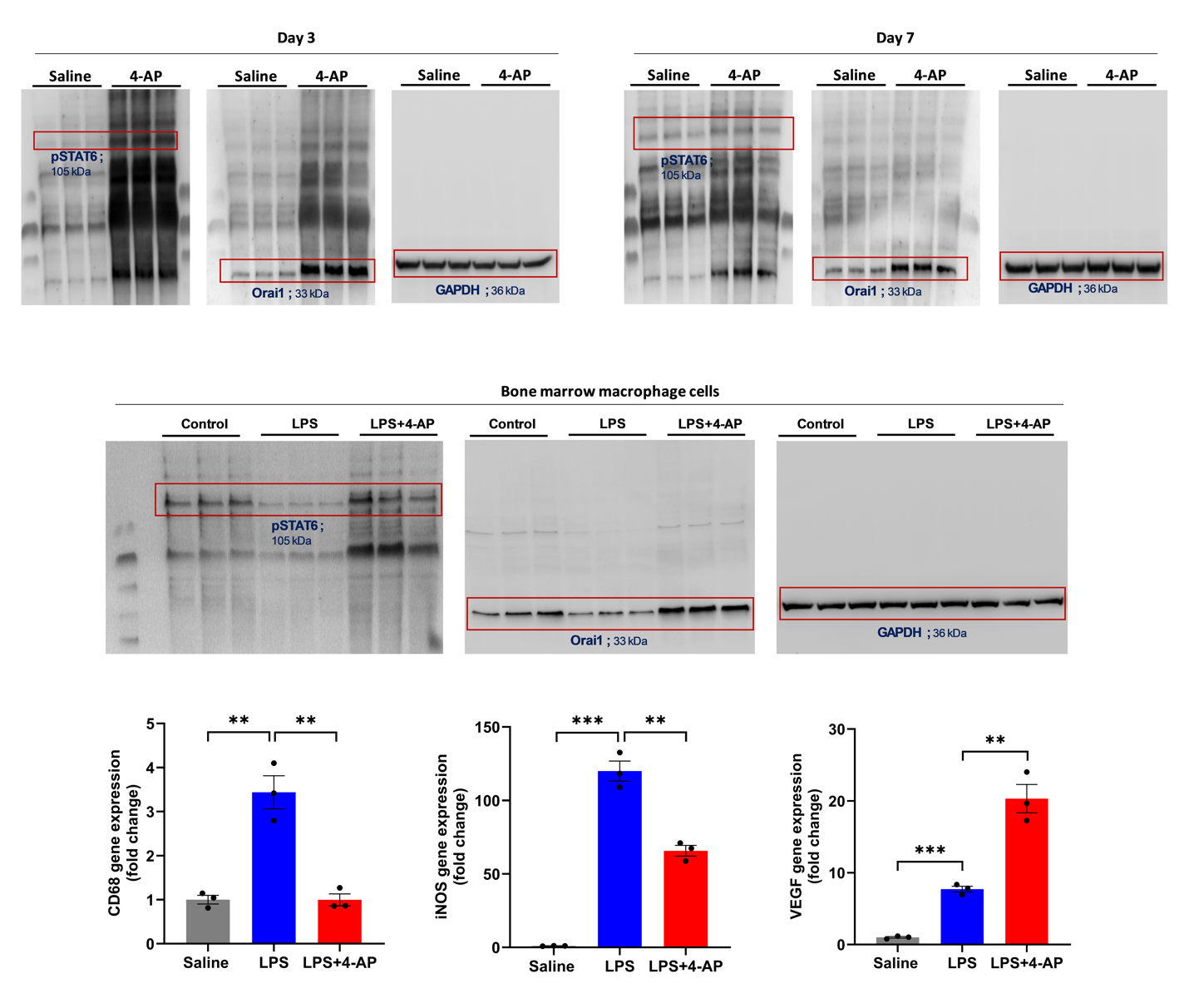
**

**Fig. 4. 4-AP augmented angiogenesis following skin burn.**

**
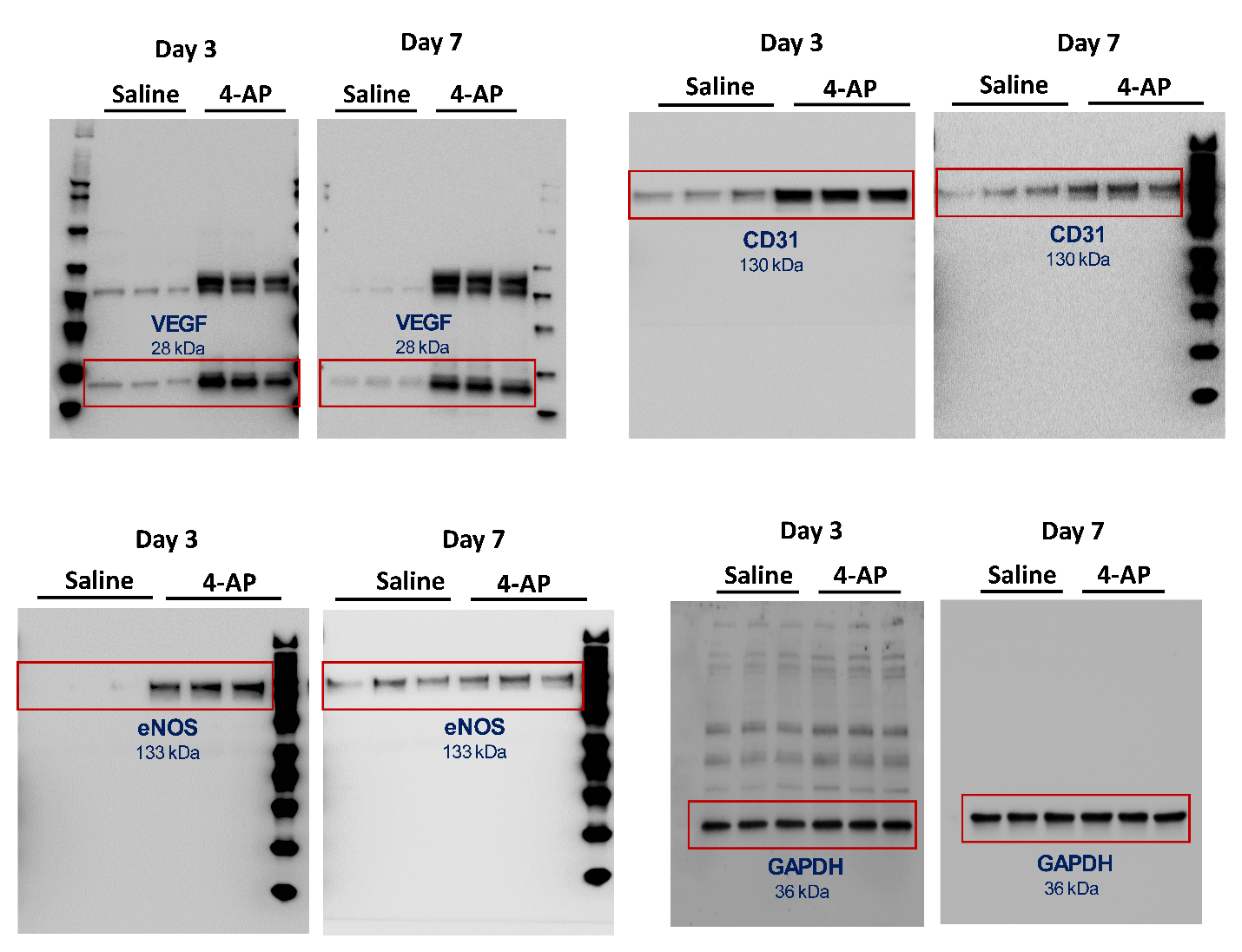
**

**Fig. 5. 4-AP attenuated pro-apoptosis and increased anti-apoptosis effects following skin burn.**

**
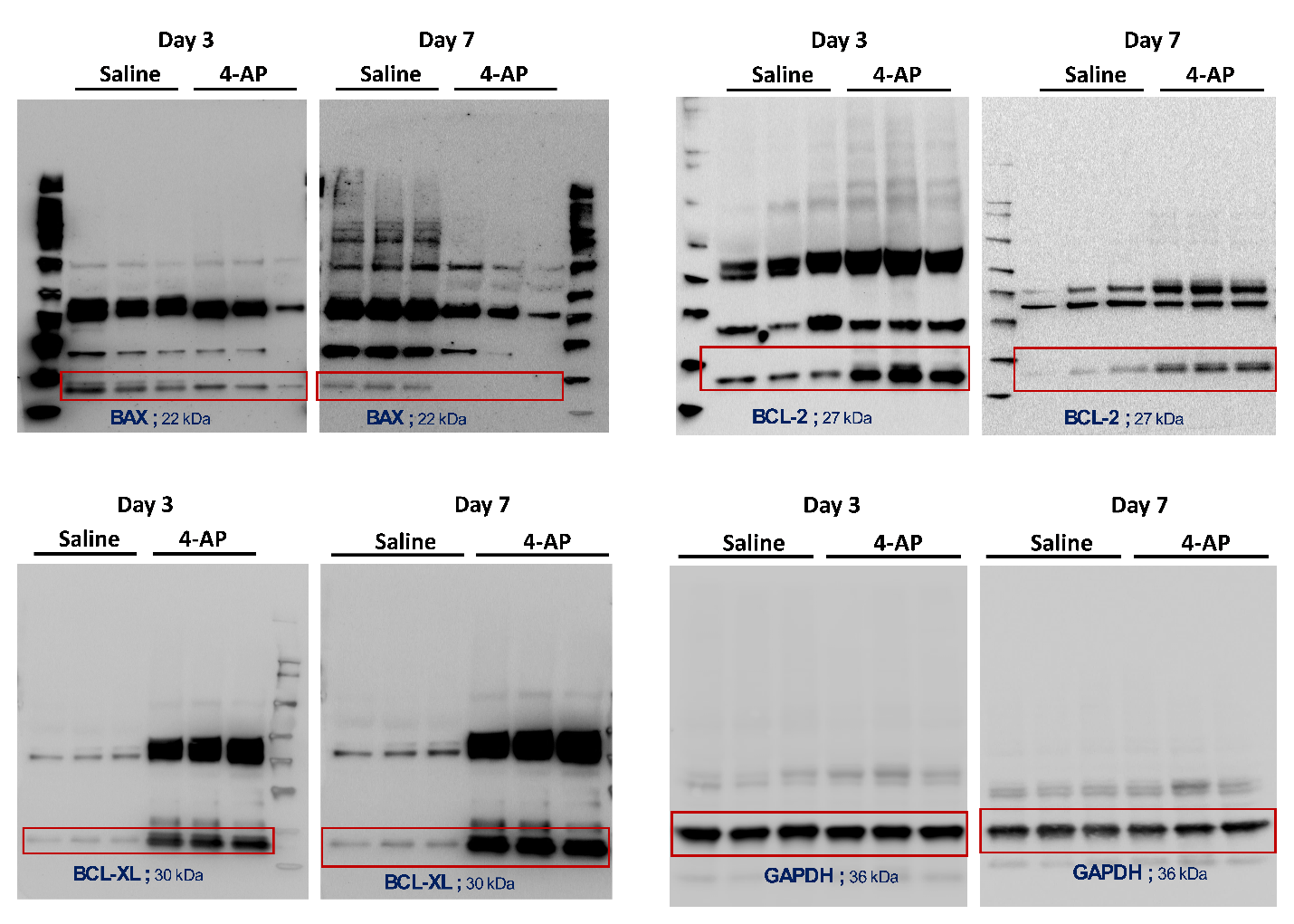
**

**Fig. 6. 4-AP accelerated re-epithelization following skin burn.**

**
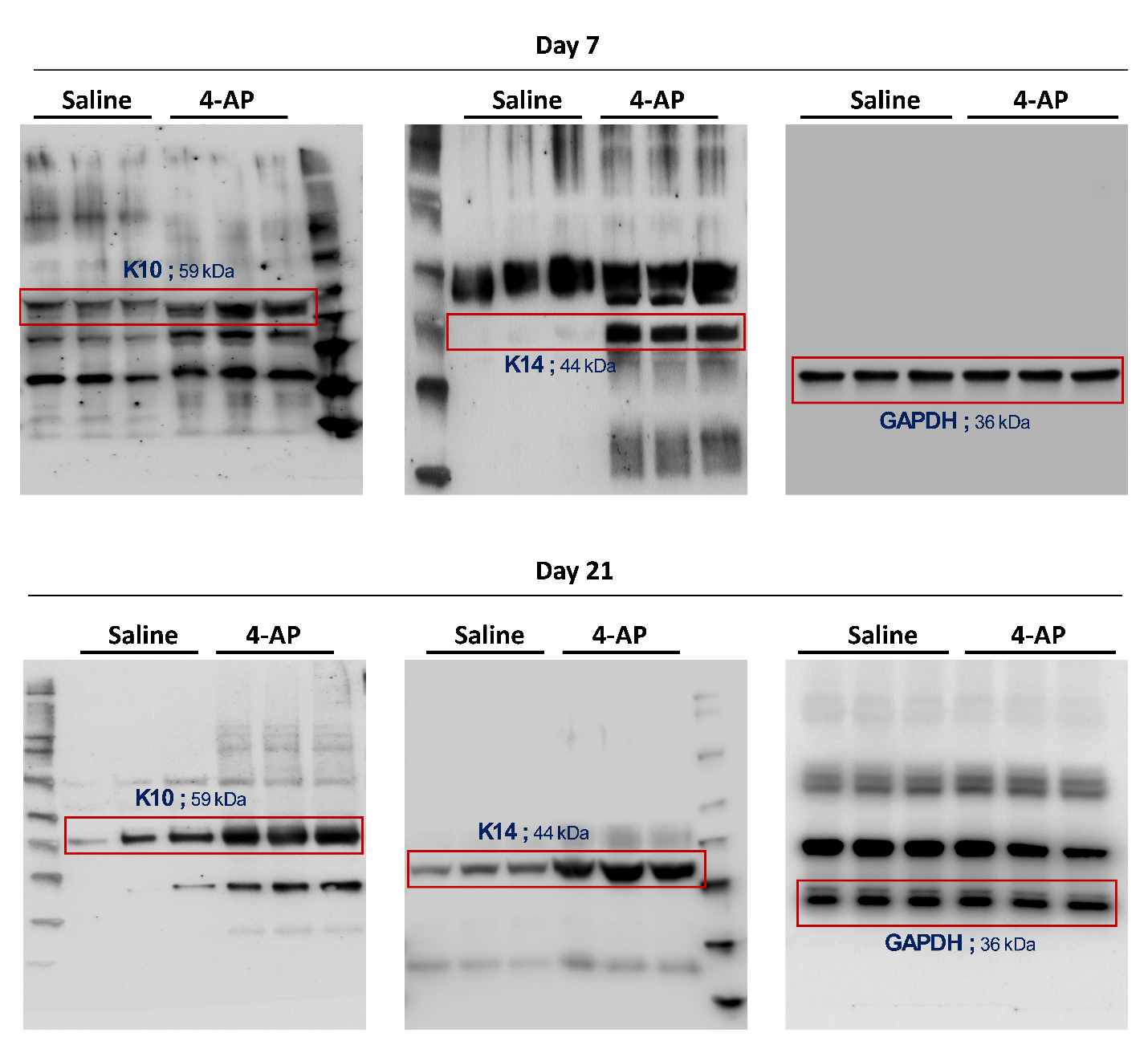
**

**Fig. 7. 4-AP promoted fibroblasts to myofibroblasts transformation following skin wound.**

**
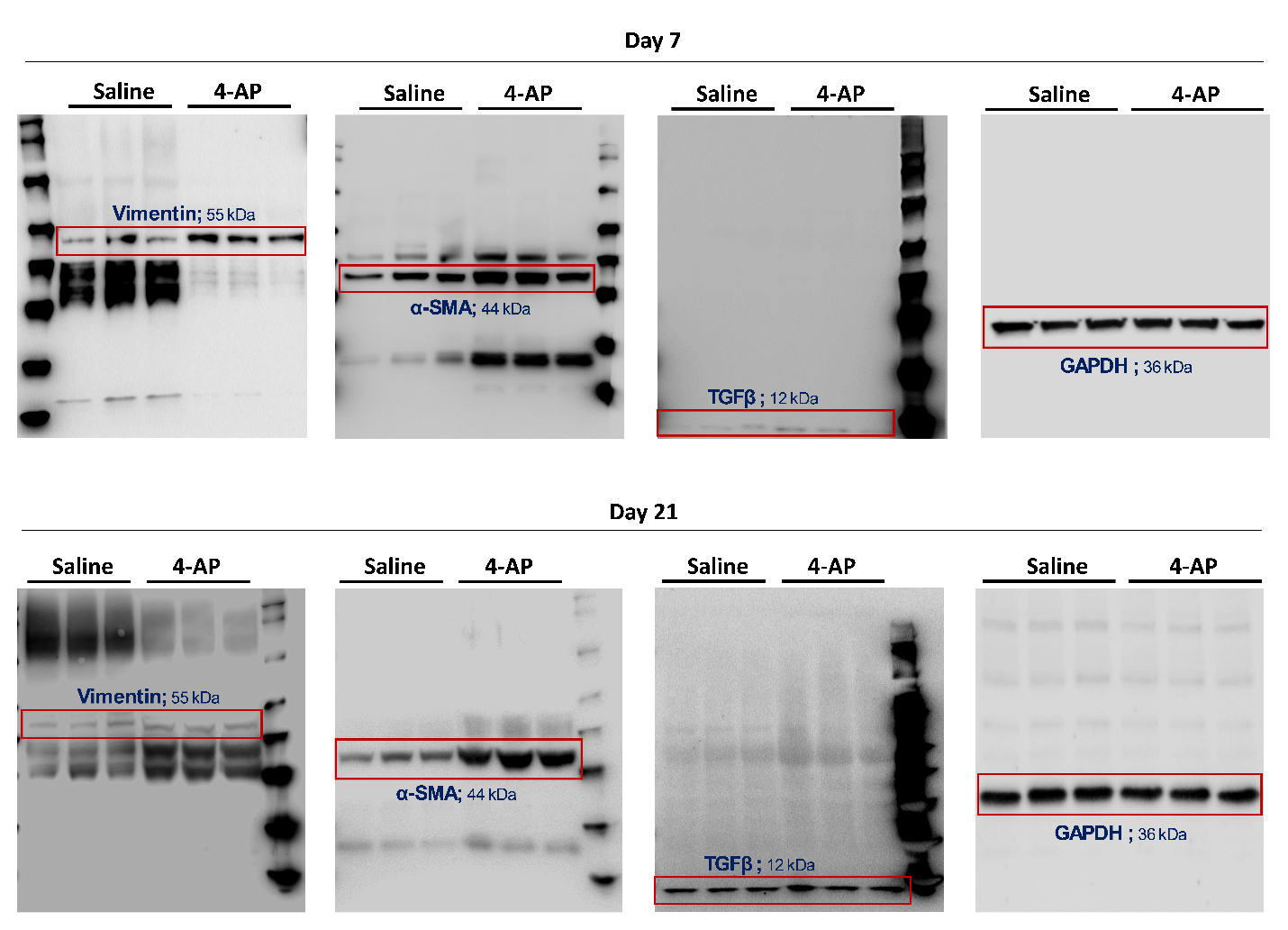
**

**Fig. 8. 4-AP advanced matrix remodeling following skin burn.**

**
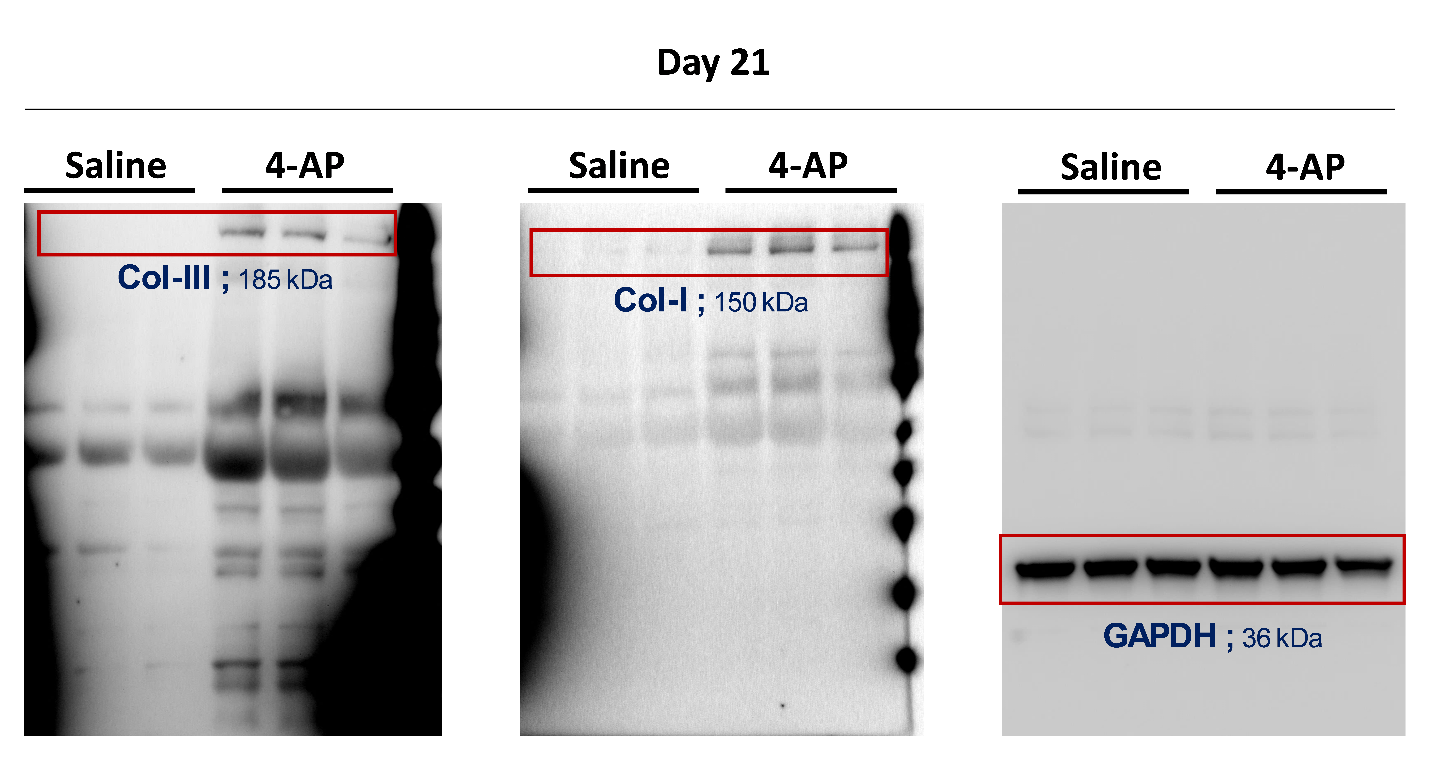
**
